# A Southeast Asian origin for present-day non-African human Y chromosomes

**DOI:** 10.1007/s00439-020-02204-9

**Published:** 2020-07-14

**Authors:** Pille Hallast, Anastasia Agdzhoyan, Oleg Balanovsky, Yali Xue, Chris Tyler-Smith

**Affiliations:** 1 Institute of Biomedicine and Translational Medicine, University of Tartu, 50411 Tartu, Estonia; 2 Wellcome Sanger Institute, Wellcome Genome Campus, Hinxton, Cambridge, CB10 1SA UK; 3 Vavilov Institute of General Genetics, Moscow, 119991 Russia; 4 Research Centre for Medical Genetics, Moscow, 115522 Russia; 5 Biobank of North Eurasia, Moscow, 115201 Russia

## Abstract

The genomes of present-day humans outside Africa originated almost entirely from a single out-migration ~ 50,000–70,000 years ago, followed by mixture with Neanderthals contributing ~ 2% to all non-Africans. However, the details of this initial migration remain poorly understood because no ancient DNA analyses are available from this key time period, and interpretation of present-day autosomal data is complicated due to subsequent population movements/reshaping. One locus, however, does retain male-specific information from this early period: the Y chromosome, where a detailed calibrated phylogeny has been constructed. Three present-day Y lineages were carried by the initial migration: the rare haplogroup D, the moderately rare C, and the very common FT lineage which now dominates most non-African populations. Here, we show that phylogenetic analyses of haplogroup C, D and FT sequences, including very rare deep-rooting lineages, together with phylogeographic analyses of ancient and present-day non-African Y chromosomes, all point to East/Southeast Asia as the origin 50,000–55,000 years ago of all known surviving non-African male lineages (apart from recent migrants). This observation contrasts with the expectation of a West Eurasian origin predicted by a simple model of expansion from a source near Africa, and can be interpreted as resulting from extensive genetic drift in the initial population or replacement of early western Y lineages from the east, thus informing and constraining models of the initial expansion.

**Electronic supplementary material:**

The online version of this article (10.1007/s00439-020-02204-9) contains supplementary material, which is available to authorized users.

## Introduction

A consensus view has emerged that the genomes of present-day human populations outside Africa originate almost entirely from a single major migration out around 50,000–70,000 years ago, accompanied or followed soon after by mixture with Neanderthals contributing ~ 2% to the genome of all non-Africans (Green et al. 2010; Mallick et al. 2016; Nielsen et al. 2017; Pagani et al. 2016). This mixture event is reliably dated from the length of the Neanderthal segments to 7000–13,000 years before the time when the Ust’-Ishim individual lived (45,000 years ago) (Fu et al. 2014). Thus, Neanderthal mixture took place 52,000–58,000 years ago, and the migration out of Africa must have occurred earlier than the mixture. The admixed population then expanded rapidly over most of Eurasia and Australia (Mallick et al. 2016; Pagani et al. 2016). As a result, people were present over much of this vast region by 50,000 years ago. The details of this initial expansion, however, remain poorly characterised. Did it follow a coastal route, an inland route, or multiple routes? Where and when did the ancestors of present-day populations begin to diverge? To what extent do present-day populations retain the genetic imprint of these early patterns? Ancient DNA studies using samples 50,000–70,000 years old could potentially provide definitive answers to these questions, but have not so far been reported because of the absence of suitable samples. Genome-wide analyses of present-day populations show a steady decrease in genetic variation with travelling distance from Africa, and have been interpreted in terms of a ‘serial founder’ model which predicts such a decrease (Prugnolle et al. 2005; Ramachandran et al. 2005). While such a pattern may have been initially established in this way, the complexity of subsequent movements and mixing events increasingly documented by ancient DNA from more recent periods (Haber et al. 2016; Yang and Fu 2018) suggests that any early pattern of population structure is unlikely to have persisted for > 50,000 years. Thus, insights into present-day autosomal genomes into the initial out-of-Africa expansion are confounded by the complexity of subsequent prehistory, suitable aDNA is not yet available, and alternative sources of information are needed. The serial founder model nevertheless provides a standard model with which alternatives can be compared.

**Fig. 1:**
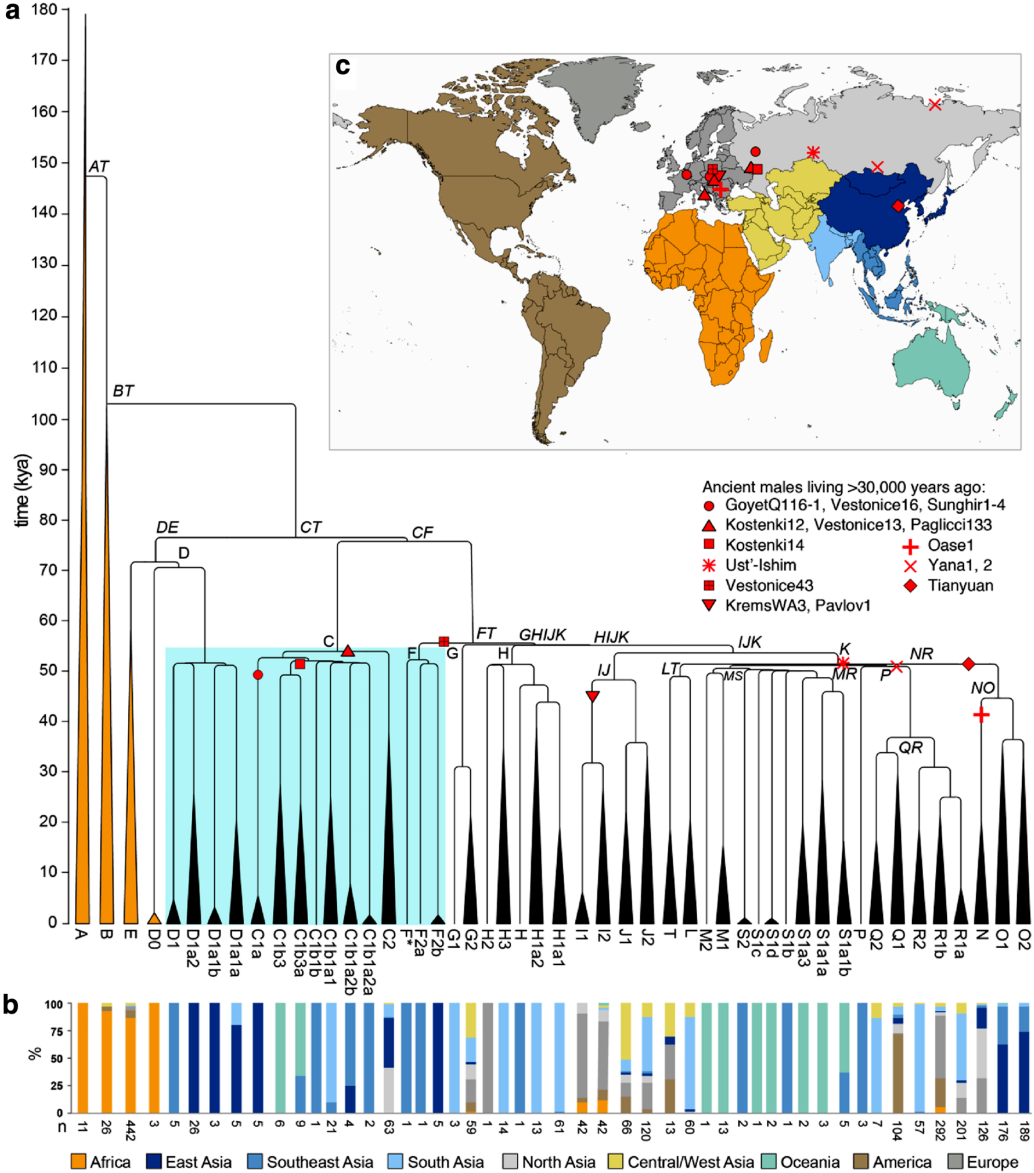
Y-chromosomal phylogeny and haplogroup distribution. **a** Maximum likelihood Y-phylogeny based on 1204 samples with branch lengths drawn proportional to the estimated times between successive splits according to BEAST analysis. Y lineages currently located in Africa are coloured gold, the others black. The key lineages of D, C and F are highlighted with a blue box. Haplogroup names indicated in italics correspond to dated splits in Supplementary Table 3. **b** Proportion of samples carrying Y lineages shown in (**a**) coloured according to geographic origin using a total of 2319 samples [1204 samples used to reconstruct the phylogeny plus 1070 non-overlapping samples from the 1000 Genomes Project (Poznik et al. 2016) and 45 samples from The Singapore Sequencing Malay Project (Wong et al. 2013)] **c** Map showing the geographic divisions used. The approximate phylogenetic locations and geographic origins of ancient male samples living more than 30,000 years ago are shown as red symbols

There is, however, one region of the genome with the potential to inform about these events in a unique way: the Y chromosome. This is because its male-specific portion provides haplotypes from which a detailed calibrated phylogenetic tree can be created (Jobling and Tyler-Smith 2017). Several such trees have been constructed independently and are all consistent in being dominated by a massive expansion of non-African Y lineages during the key interval of 50,000–60,000 years ago starting from a single haplogroup designated CT (Hallast et al. 2015; Karmin et al. 2015; Poznik et al. 2016; Wei et al. 2013) (see Fig. 1 for haplogroup designations). Taking into account a rare African D0 lineage and the timeframe summarized above, we have argued (Haber et al. 2019) that the initial splits within CT are likely to have occurred in Africa before the exit, and that three lineages, C, D and FT, were carried out by the ancestors of present-day non-Africans. Each of these three lineages subsequently expanded: C and D moderately, and FT massively. We, therefore, set out to re-examine the early divergences within these three lineages to investigate the insights they can provide into male history and perhaps human history more generally in this early period.

## Results and discussion

We assembled available sequences of C, D and FT lineages from worldwide surveys ensuring that common lineages were represented (Bergstrom et al. 2020; Karmin et al. 2015; Mallick et al. 2016; Meyer et al. 2012; Poznik et al. 2016), and supplemented them with additional sequences from known rare lineages potentially relevant to early divergences, specifically, Australian C (Bergstrom et al. 2016; Mallick et al. 2016), West African D0 (Haber et al. 2019), Andamanese D (Mondal et al. 2017), and F chromosomes from China (Mallick et al. 2016), Vietnam (Poznik et al. 2016) and Singapore (Wong et al. 2013): 1204 sequences in all. We then focussed on the phylogenetic structure of the early divergences within these three lineages, and their geographical distributions revealed by ancient DNA and present-day analyses.

**Fig. 2:**
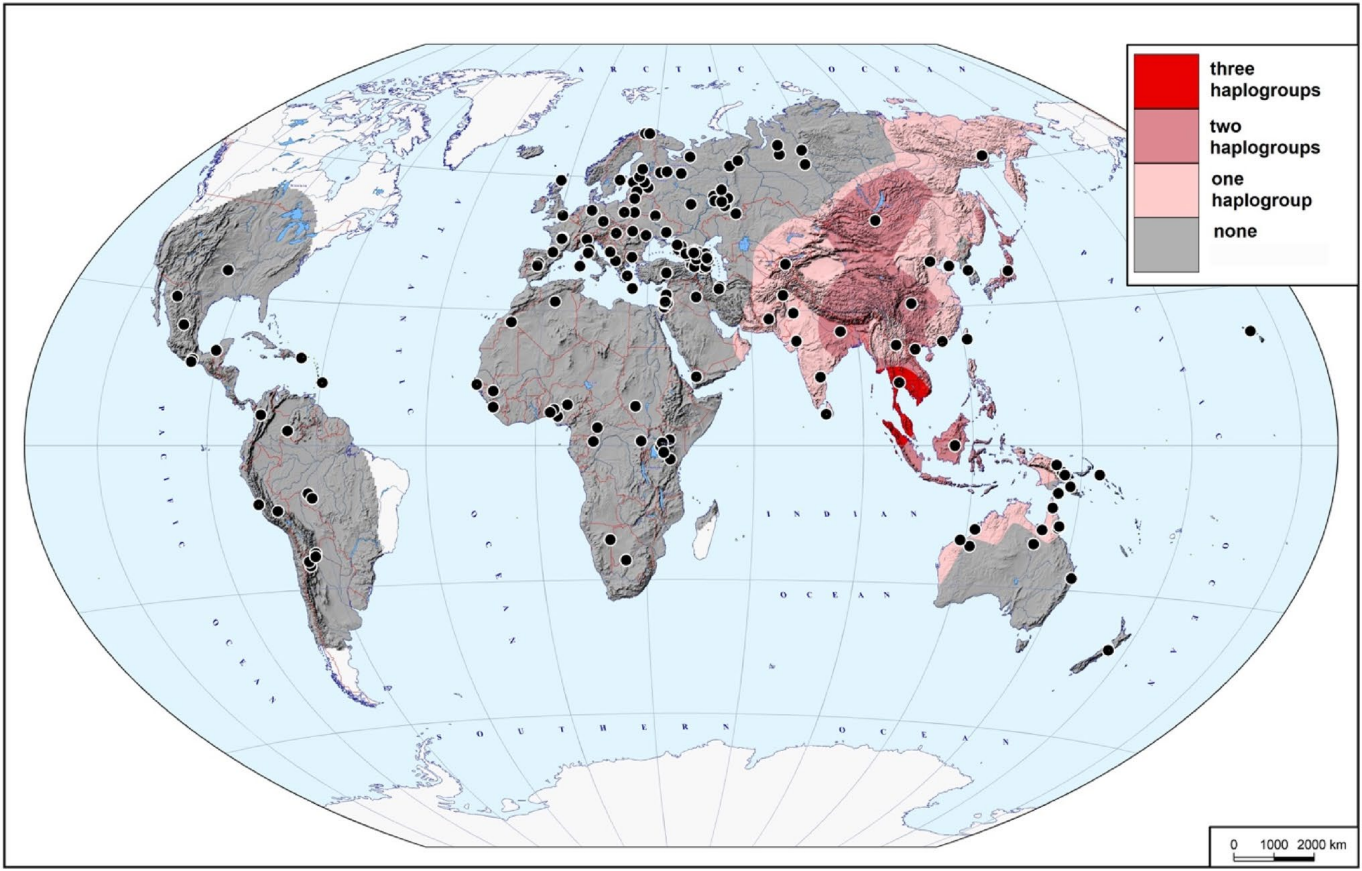
Presence of haplogroups C, D and F in 2302 present-day samples. The map demonstrates how many of the three haplogroups of interest (none, one, two, or all three) were found in different areas of the Old World and Near Oceania. Black dots indicate the locations of the studied populations

The resulting Y-chromosomal tree (Fig. 1) depicts 50 lineages, with the African lineages (gold) represented only by the four major African haplogroups without including their subsequent branches, but with the non-African lineages represented more fully to include all those originating before 45,000 years ago and found in the sample of present-day Y chromosomes examined, together with some of the more abundant recent lineages. As expected from previous analyses, this phylogeny shows that the three initial lineages C, D and FT each underwent initial rapid expansions soon after 54,000 (95% highest posterior density [HPD], 44,400–64,100) years, so that by 50,000 (95% HPD, 43,700–64,100) years ago there were seven branches within C, 5 within D and 18 within FT (30 non-African lineages in all); by 45,000 (95% HPD, 40,200–64,100) years ago the number of branches within FT had increased to 24 (36 in all). The branching patterns, together with the present-day locations of the lineages derived from an analysis of 2319 sequences, provide insights into possible locations of the early expansions. Lineage C split into two, C1 and C2; C1 lineages are found today only in East, Southeast and South Asia plus Oceania, while C2 lineages are more widespread and are now found in East and South Asia and also North and Central/West Asia (Fig. 2, Supplementary Fig. 1). D lineages are entirely confined to East and Southeast Asia. FT lineages now have a worldwide distribution, but the earliest split was into F and GHIJK; F is known only from East and Southeast Asia (Fig. 1, Supplementary Fig. 1), while GHIJK and its descendants are found worldwide. These descendant lineages themselves often have more continent-specific distributions, but 14/15 GHIJK lineages originating before 50,000 (95% HPD, 43,700–63,300) years ago have distributions that include East, Southeast or South Asia, apart from a few that are specific to Oceania (Fig. 1). Only one (H2, represented by a single sample) is specific to Europe, and none to the region adjoining the likely exit routes from Africa, in the terminology used Central/West Asia, where less than half are now present in the samples examined.

No ancient Y-chromosomal data earlier than 45,000 years ago have been reported, but 21 Asian or European males living 30,000–45,000 years ago are documented, and for 18 of them assignments to C, D or FT have been reported (Fig. 1, Supplementary Fig. 1, 2, Supplementary Table 1) (Fu et al. 2014, 2015, 2016; Seguin-Orlando et al. 2014; Sikora et al. 2017, 2019; Yang et al. 2017). Ten belong to the C lineage, six from North Asia and four from Europe. The remaining eight belong to FT, three from North Asia, one from East Asia and four from Europe. Although the data are limited, two conclusions can be drawn. First, none of the ancient samples carry Y lineages outside the 30 represented in Fig. 1 at 50,000 years ago. Second, C lineages (both C1a and C1b), now confined to East, Southeast and South Asia plus Oceania, were more widespread 30,000–40,000 years ago, including in Europe where they persisted until after 8000 years ago (Mathieson et al. 2018), although they have now been replaced in Europe by other lineages.

**Fig. 3:**
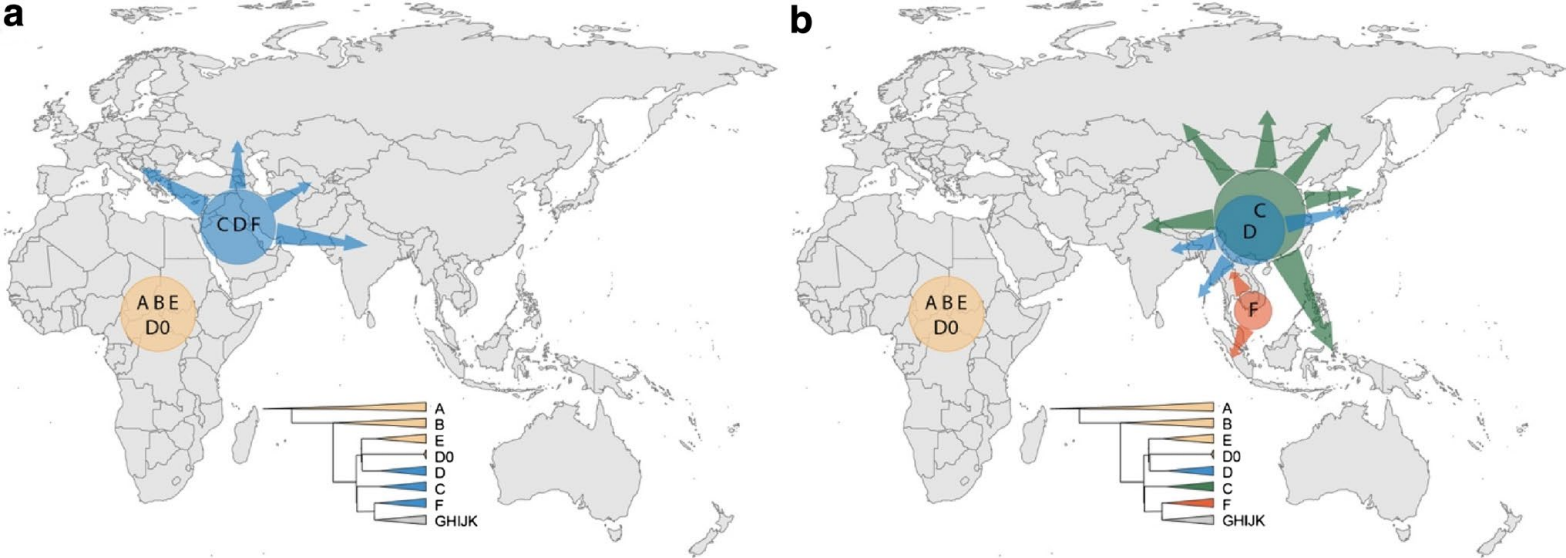
According to serial founder model, the earliest-branching non-African lineages are expected to expand and be present closer to Africa (**a**), but instead have expanded in East or Southeast Asia (**b**). Simplified Y tree is shown as reference for colours

In a simple model of gradual human expansion from Africa to Asia and Oceania without subsequent continental-scale reshaping, we would expect the initial divergences in the Y-chromosomal phylogeny to have occurred in geographical locations close to Africa, and the present-day Y-chromosomal phylogeography to reflect this history by showing the presence of the early-diverging lineages within C, D and FT now being located geographically in Central/West Asia (Fig. 3a), with lower lineage diversity further east. In stark contrast, the observed distributions of these lineages all lie further to the east, suggesting that a simple model of this kind cannot explain the observed present-day data (Fig. 3b, Supplementary Fig. 3), a discrepancy we discuss further below.

The phylogeny of maternally inherited mitochondrial DNA (mtDNA), like that of the Y chromosome, also retains information from 50,000 to 70,000 years ago, although female-specific and with less detail because of its shorter length. Nevertheless, it provides a useful comparison. Outside Africa, the initial split inferred from a combination of ancient and present-day sequences was between lineages M and pre-N, with divergence within M dated to 44,000–55,000 years ago and within N to 47,000–55,000 years ago (Posth et al. 2016). Present-day geographical distributions of mtDNAs are less specific than Y chromosomes, and both of these major lineages are widespread worldwide, although M is absent from present-day Europeans with the exception of recent migrations (Gonzalez et al. 2007). Nevertheless, M was present in early Europeans until at least 28,000 years ago; moreover, the first branch within the pre-N/N lineage is between the pre-N mtDNA carried by Oase1 from Romania dating to 40,000 years ago (who, incidentally, showed increased Neanderthal admixture) and the remaining worldwide N mtDNAs. mtDNA thus shares with the Y chromosome a history of continental-scale change (loss of M from Europe), in the case of mtDNA dated to after 28,000 years ago. In addition, mtDNA N demonstrates the phylogeographic pattern expected from a simple expansion model, with its earliest divergence in the west.

How then can the present-day Y-chromosomal phylogeography be reconciled with an out-of-Africa expansion? It is well established that all known present-day Y-chromosomal lineages trace back to Africa at some point in human history (Jobling and Tyler-Smith 2017), but the current work demonstrates that the deepest rooting C, D and FT lineages now seen outside Africa are found in East/Southeast Asia. Without support from additional ancient DNA samples, it is difficult to make claims about the geographic origins of these deep-rooting lineages; however, this difficulty does not change the observation about their current location. The default explanation for the observed patterns is perhaps that the initial divergences within the Y-chromosomal phylogeny did indeed occur in the west, but that the deepest rooting lineages have now been lost from this part of the world, consistent with the lack of genetic continuity in West Eurasia seen in autosomal aDNA and the presence of Y haplogroup C lineages in West Eurasia until ~ 8000 years ago (Mathieson et al. 2018). In principle, this could be because C, D and F lineages all migrated east, together with some GHIJK lineages, leaving only GHIJK lineages in the west; or more plausibly that C, D and F were lost by genetic drift in the west, but not in the east. The first scenario would imply unprecedented levels of male-structured migration, and would be difficult to reconcile with subsequent divergences within GHIJK during the next few thousand years, whereby some of the descendent lineages such as G1, H1 and H3 would also need to have migrated east in a male-structured way. The second scenario is not easy to reconcile in a simple way with the inference that genetic effective population sizes have been lower in East Asia than in Europe (Gutenkunst et al. 2009; Kelleher et al. 2019), so less genetic drift is expected in the west. Further explanations should, therefore, also be considered; one such is that initial western Y chromosomes have been entirely replaced by lineages from further east (Fig. 3), perhaps on more than one occasion. This is supported by the observed patterns of early-diverging lineages of C, D and FT now being located in East and Southeast Asia, and, according to our present-day dataset of surviving lineages, the more likely origin of GHIJK in the east (Fig. 1). Formally, another explanation could be that selection has acted, for example, to favour the FT lineage to different extents in different regions, but positive natural selection has not been documented on the human Y chromosome (Jobling and Tyler-Smith 2017) and there are no candidate coding variants reported among annotated protein-coding genes (Poznik et al. 2016), so this seems unlikely. Nevertheless, the possible explanations for observed patterns cannot be reliably differentiated at present. Until aDNA data earlier than 45,000 years ago are available, future studies using spatial simulations with models that are able to adequately capture the complexity of the human past may help to explain the observed patterns in the present-day human Y-chromosomal data.

Ancient DNA studies are beginning to show some of the true complexity of human genetic history, including providing evidence for large-scale intercontinental movements in the last 30,000 years or so (Fu et al. 2014, 2015, 2016; Seguin-Orlando et al. 2014; Sikora et al. 2017, 2019; Yang et al. 2017). The out-of-Africa model requires major intercontinental movements 40,000–60,000 years ago, as well as later expansion into the Americas. From these perspectives, it is perhaps more likely that large-scale movements have continued throughout human prehistory than not, and replacement from the east is thus an explanation to consider. Ultimately, the prehistory of this period must encompass fossil, archaeological and multiple forms of genetic data, and reconcile them into a coherent overall understanding. The unique genetic properties of the Y chromosome may offer insights into movement during an early period that is currently difficult to investigate in other ways and provide a glimpse of this prehistory.

## Materials and methods

### Data

Y-chromosomal data from high-coverage whole-genome sequenced samples were combined from the following publicly available or published datasets: the Simons Genome Diversity Project (SGDP) (Mallick et al. 2016), Polaris (https://github.com/Illumina/Polaris), the Human Genome Diversity Project (HGDP) (Bergstrom et al. 2020; Meyer et al. 2012), the Andaman Islands samples (Mondal et al. 2017), haplogroup D0 samples from Nigeria and additional haplogroup D samples from Tibet (Haber et al. 2019), Australian haplogroup C samples (Bergstrom et al. 2016) and a haplogroup F* Singapore Malay sample SSM072 (Wong et al. 2013). Fifty low-coverage whole-genome sequenced samples from the 1000 Genomes Project dataset (Poznik et al. 2016) were included to represent some of the deep-rooting lineages of haplogroups A, C, F, and H that were not present in other datasets. Additionally, 303 publicly available samples (Karmin et al. 2015) sequenced at Complete Genomics (CG) were included.

The HGDP, SGDP, Simons, Polaris and Tibetan samples had been mapped to GRCh38, and the Australian, haplogroup D0 and 1000 Genomes Project samples to GRCh37. The reads mapping to the Y chromosome from GRCh37-mapped haplogroup D0, Malay and Andaman Islands samples were extracted using picard (v2.7.2), re-mapped to the GRCh38 using bwa mem (v0.7.17) (Li and Durbin 2009), followed by duplicate removal using samtools (v1.8).

The genotypes of samples mapped to GRCh38 were jointly called using bcftools (v1.8) with minimum base quality 20, mapping quality 20 and defining ploidy as 1, using the 10.3 Mb of chromosome Y sequence previously defined as accessible to short-read sequencing (Poznik et al. 2013). Similarly, samples mapped to GRCh37 were jointly called using identical parameters. The calls were filtered as follows: removing single nucleotide variants (SNVs) within 5 bp of an indel (SnpGap) and removing indels. The genotypes of high-coverage samples with an overall mean read depth on chromosome *Y* ≥ 12 × were filtered for minimum read depth of 3, samples with lower mean read depth for minimum read depth of 2, except that no minimum read depth filter was applied to the 1000 Genomes Project low-coverage samples. Additionally, if multiple alleles were supported by reads, then the fraction of reads supporting the called allele should be ≥ 0.85; otherwise, the genotype was converted to missing data. The CG dataset was obtained as a GRCh37 all-site vcf file, where all genotypes with the CG-specific VQLOW quality tag had been converted to missing data. All GRCh37-based vcf files were then merged using bcftools, lifted over to GRCh38 using picard followed by merging with the rest of GRCh38-based data. High-coverage samples with ≥ 5% of missing data across all sites and sites with ≥ 3% of missing calls across samples were removed using vcftools (v0.1.14). Two samples (CongPy6 and ISR07) from the CG dataset were later removed due to unusually long terminal branches. After filtering, a total of 10,191,767 sites remained, including 86,080 variant sites (49,799 singletons) (Supplementary Dataset 1).

The final dataset includes 1208 samples: 610 from the HGDP, 95 from the SGDP, 126 from the Polaris dataset, 13 Australian aboriginal samples, five samples from the Andaman Islands, 7 haplogroup D samples, 301 CG samples, 1 Singapore Malay and 50 low-coverage samples from the 1000 Genomes project (Supplementary Table 2).

Two overlapping samples (HG03100 and HG00190) between the Simons and Polaris datasets and two duplicate samples in the CG dataset (Murut5 and Komi2) were retained as internal controls, making it a total of 1204 independent individuals.

In addition, 1070 non-overlapping samples from the 1000 Genomes Project (Poznik et al. 2016) and 45 samples from The Singapore Sequencing Malay Project (Wong et al. 2013) (Supplementary Tables 4, 5) were included in the phylogeographic analysis using previously defined Y lineage information.

### Phylogenetic tree construction and dating

The maximum likelihood Y-phylogeny including 1208 samples and 86,080 variant sites was inferred using RAxML v8.2.10 with the GTRGAMMA substitution model (Stamatakis 2014). The tree was visualized using the FigTree software (v1.4.4) (http://tree.bio.ed.ac.uk/software/figtree/) with midpoint rooting (Supplementary Fig. 4).

The ages of the internal nodes in the phylogenetic tree were estimated using both the *ρ* statistic (Forster et al. 1996) and the coalescent-based method implemented in BEAST (Drummond and Rambaut 2007; Drummond et al. 2005) using only the high-coverage genomes (Supplementary Table 2).

The ρ statistic was estimated as described (Bergstrom et al. 2016). Briefly, the pairwise divergence estimates were obtained from the final all-site vcf, ignoring sites with missing genotypes in either of the samples. If multiple samples were available in a given clade, then per-pair divergence estimates were averaged across them. The divergence times in units of mutations per site were converted to units of years by applying a point mutation rate of 0.76 × 10^−9^ mutations per site per year (Fu et al. 2014). The 95% confidence intervals of the divergence times were estimated using the uncertainty of the mutation rate (0.67–0.86 × 10^−9^) (Fu et al. 2014). To reduce the computational cost, if either group of descendants of the node to be dated contained more than 100 samples, then 1/3 of randomly selected samples were used to obtain the pairwise divergence estimates.

To reduce the computational cost of running BEAST, a smaller dataset containing 332 samples was used for dating. Samples were selected to represent the major branches in the phylogenetic tree and also all the haplogroup C, D and F samples (Supplementary Table 2).

An initial maximum likelihood phylogenetic tree was constructed using RAxML with a set of 50,686 variant sites, then using this as a starting tree for BEAST (v1.8.4). Markov chain Monte Carlo samples were based on 131 million iterations, logging every 1000 iterations. The first 10% of iterations were discarded as burn-in. Eight independent runs were combined using LogCombiner. A constant-sized coalescent tree prior, the HKY substitution model, accounting for site heterogeneity (gamma) and a strict clock with a substitution rate of 0.76 × 10^−9^ (95% confidence interval: 0.67 × 10^−9^–0.86 × 10^−9^) single nucleotide mutations per bp per year (Fu et al. 2014) was used. A prior with a normal distribution based on the 95% confidence interval of the substitution rate was applied. Only the variant sites were used, but the number of invariant sites was defined in the BEAST xml file. A summary tree was produced using TreeAnnotator (v1.8.4) and visualized using the FigTree software (Supplementary Fig. 5).

### Y haplogroup nomenclature

The Y haplogroups of each sample were predicted from the all-site vcf file with the yHaplo software (https://github.com/23andMe/yhaplo) using a version where the marker coordinates in the relevant input files had been replaced to correspond to the GRCh38 assembly (Bergstrom et al. 2020). The identified terminal SNV for each sample was used to update the haplogroup name to correspond to the International Society of Genetic Genealogy nomenclature (ISOGG, https://isogg.org, v03.10.19) (Supplementary Table 2). The exceptions were haplogroup C, D and K*/M samples for which the states (ancestral or derived) of all haplogroup-specific markers included in ISOGG v03.10.19 database were checked and the haplogroup name updated according to the most terminal SNV in derived state. Additionally, for haplogroup D0 samples, the original nomenclature (Haber et al. 2019) was followed (Supplementary Table 2). For the haplogroup F samples, the following nomenclature is suggested to correspond to the phylogenetic tree: sample HG02040 to be defined as F*, SSM072 as F2a and the five Lahu samples (HGDP01317, HGDP01318, HGDP01320, HGDP01321 and HGDP01322) as F2b (defined as F2 according to the ISOGG database).

### Cartographic analysis

The combined dataset of 2302 *Y* chromosomes from 269 populations with geographic coordinates of origin available (Supplementary Table 5) was used to create the distribution maps of three key haplogroups (C, D, and F). As many populations were represented by very few samples, data on neighbouring populations were merged to achieve the average sample size of approximately 50 in the areas with non-zero frequencies of the haplogroups of interest (Supplementary Table 5). The GeneGeo software (Balanovsky et al. 2011; Koshel 2012) was used with the generalized Shepard’s method, weight function 3 and radius of influence of 2000 km to create the grids of interpolated values. The frequency distribution maps (not shown) were created as well as the maps demonstrating presence or absence of a haplogroup (Supplementary Fig. 3). In each node of the cartographic grid, the values of these three haplogroup presence maps have been summarized, and combined into a single map (Fig. 2) indicating how many of the three haplogroups of interest (none, one, two, or all the three) were found in different areas of the Old World.

## Electronic supplementary material

Below is the link to the electronic supplementary material.

Supplementary material 1 (DOCX 1582 kb)

Supplementary material 2 (XLSX 141 kb)

Supplementary material 3 (PDF 757 kb)

Supplementary material 4 (PDF 269 kb)

Supplementary material 5 (GZ 330 mb)

## Data Availability

Final filtered vcf containing variant sites for 1208 samples is available as Supplementary Data file 1.
