# Supplementary material for: A Southeast Asian origin for present-day non-African human Y chromosomes
Source: Hum Genet. 2020 Jul 14;140(2):299–307. doi: 10.1007/s00439-020-02204-9 (PMC7864842; doi:10.1007/s00439-020-02204-9)
Supplement: Supplementary file 1 — Supplementary material 1 (DOCX 1582 kb) [file 439_2020_2204_MOESM1_ESM.docx]

**
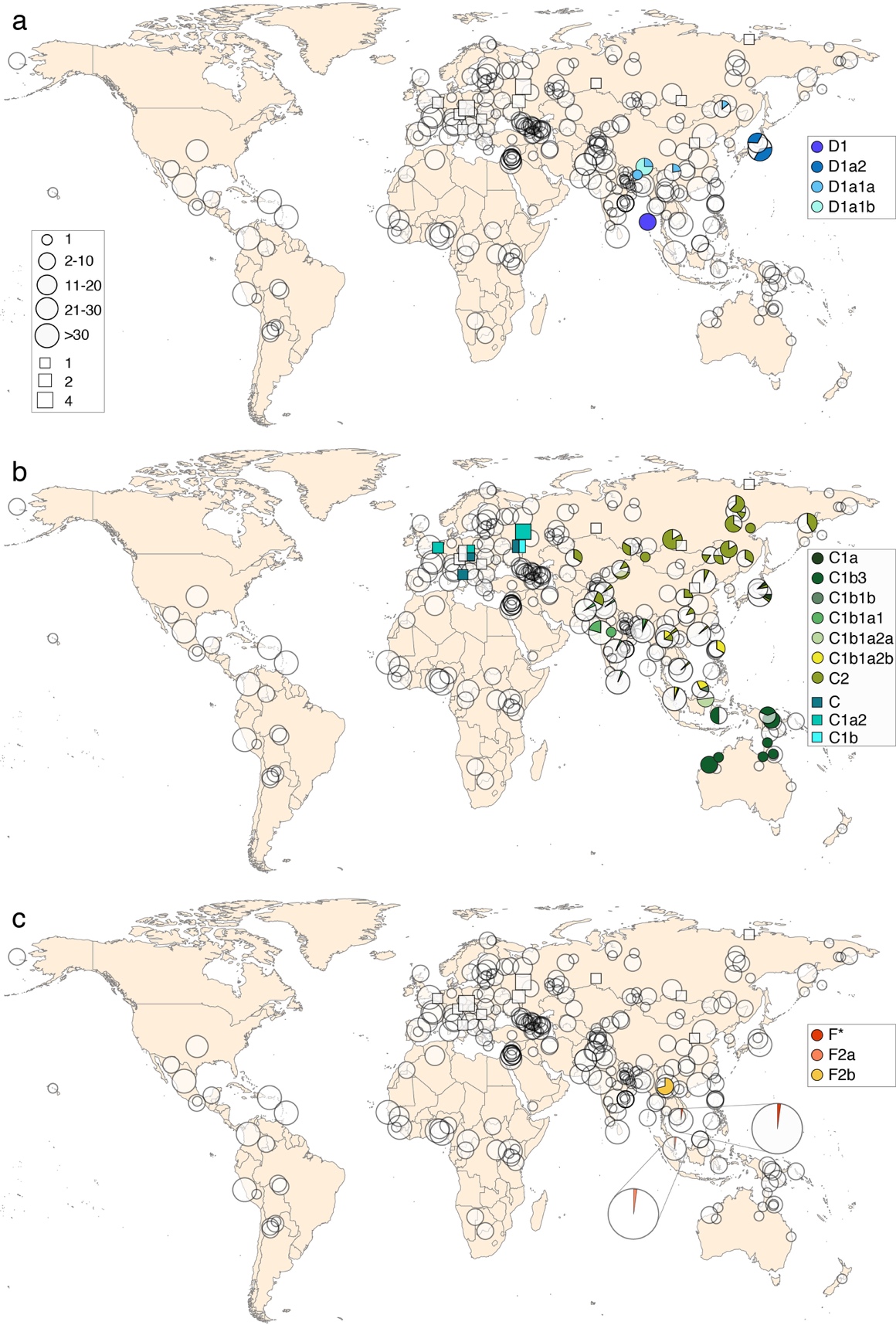
**

**Supplementary Figure 1**. Geographic distribution of males carrying haplogroups D **(a)**, C **(b)** and F **(c).** The geographic origin and approximate sample sizes are shown for 2302 modern samples as circles (for 17 samples from the CG dataset the exact geographic coordinates were not available). Ancient male samples living more than 30,000 years ago are shown as squares, the number of samples corresponding to the size of the square.

**
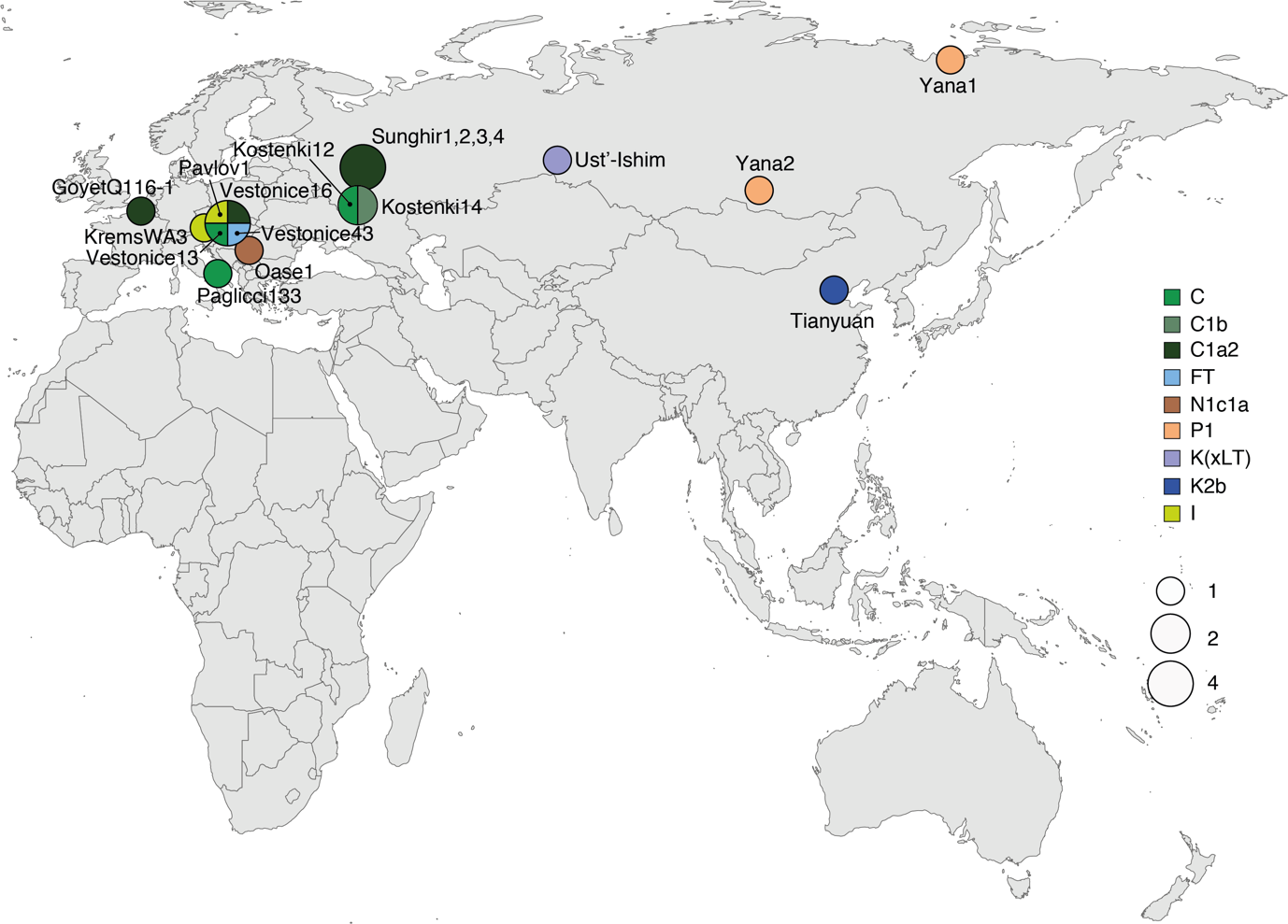
**

**Supplementary Figure 2**. Geographic distribution of Y lineages among ancient male samples living more than 30,000 years ago with original sample names shown.


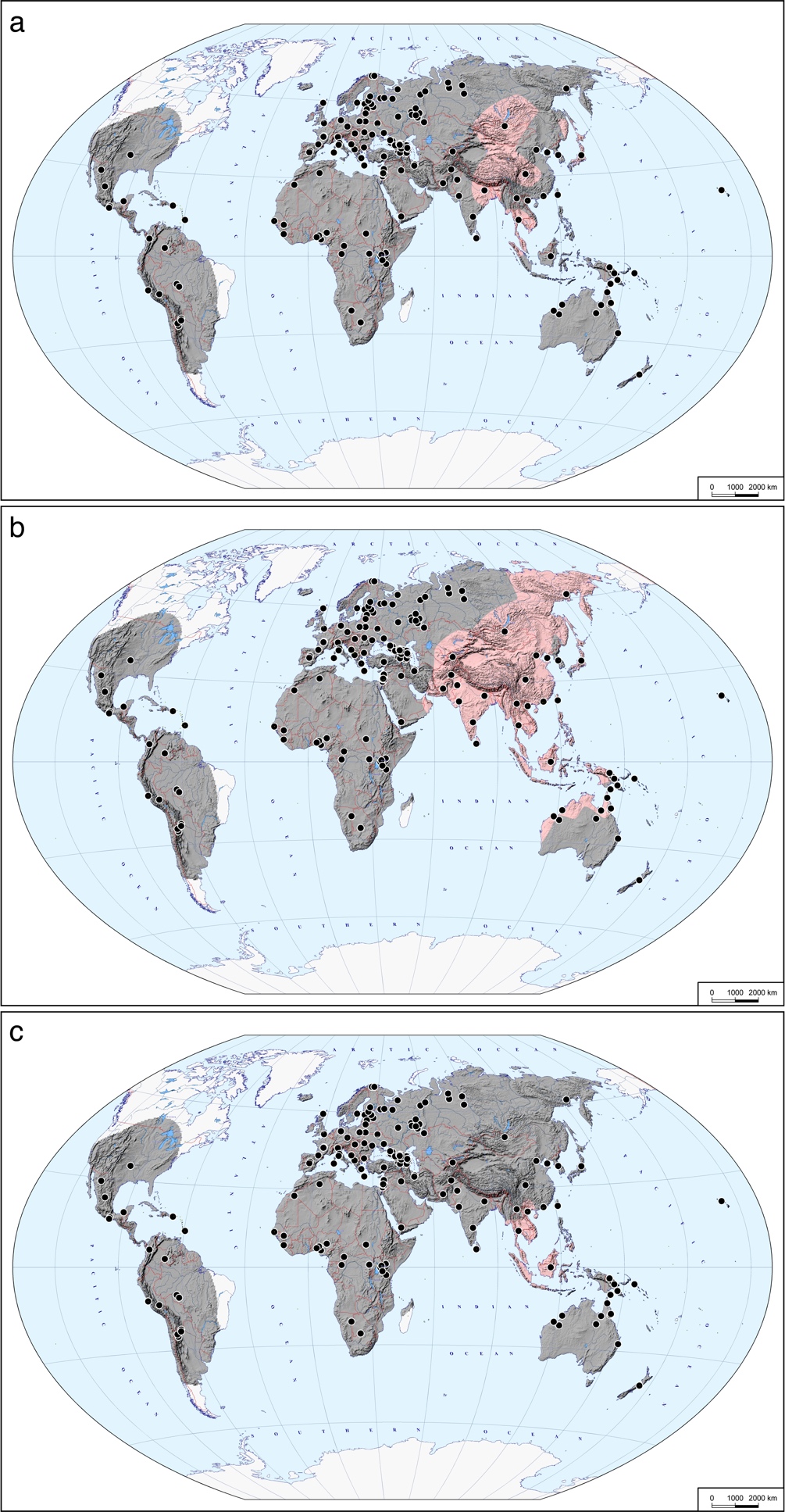


**Supplementary Figure 3**. Distribution of haplogroups D **(a)**, C **(b)** and F **(c)** in 2302 samples. Pale red and grey colours indicate the presence or absence of the haplogroup, respectively. Black dots correspond to the geographic origins of the studied populations.

[Included as a separate file]

**Supplementary Figure 4**. Maximum likelihood Y-chromosome phylogeny including all 1208 samples used in the study. The sample name in the tree shows the sample ID, population, country of origin and Y haplogroup separated by underscores and is coloured according to the geographic origin of the sample.

[Included as a separate file]

**Supplementary Figure 5**. Y chromosome phylogeny based on BEAST analysis of 332 samples. The sample name in the tree shows the sample ID, population, country of origin and Y haplogroup separated by underscores and is coloured according to the geographic origin of the sample. Age and posterior support estimates for main clades are reported in Supplementary Table 3.
