# Supplementary material for: A Southeast Asian origin for present-day non-African human Y chromosomes
Source: Hum Genet. 2020 Jul 14;140(2):299–307. doi: 10.1007/s00439-020-02204-9 (PMC7864842; doi:10.1007/s00439-020-02204-9)
Supplement: Supplementary file 3 — Supplementary material 3 (PDF 757 kb) [file 439_2020_2204_MOESM3_ESM.pdf]

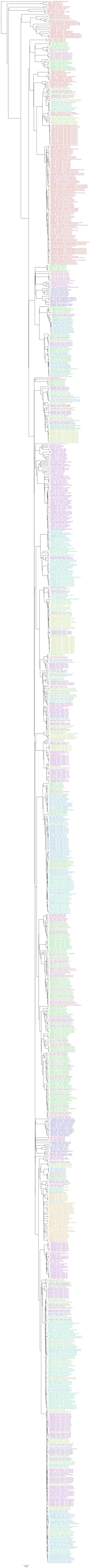
0.002
